# Understanding cognitive differences in the effect of digitalization on ambidextrous innovation: Moderating role of industrial knowledge base

**DOI:** 10.3389/fpsyg.2022.983844

**Published:** 2022-09-13

**Authors:** Qiang Xu, Hanlin Liu, Yi Chen, Kexin Tian

**Affiliations:** School of Management, Zhejiang University of Technology, Hangzhou, China

**Keywords:** cognitive differences, digitization level, industrial knowledge base, ambidexterity, exploratory innovation, exploitative innovation, knowledge-based view

## Abstract

A number of existing researches agree that digitalization would facility firms to launch ambidextrous innovations. Digitalization is not only about technological change, but more importantly, the reshaping of the firms’ knowledge structure and routines to percept and integrate knowledge. Thus, some researchers suggest that whether firms could benefit from digitalization varies across firms and industries, since innovation in different firms and industries relies on differentiated level of cognitive and reasoning of knowledge. However, existing studies mainly focus on exploring the firm-level differences, and leave the industry-level difference underdeveloped. In response, this study integrates knowledge-based view to examine how a firm’s digitalization affects ambidextrous innovation, and further explore the conjoint effect of industrial knowledge bases—the knowledge base of the industry the firm is located in—on the relationship between firm digitalization level and ambidextrous innovation. This study uses Python to conduct text mining of firms’ annual reports, and obtains data of 394 listed companies from the year 2014 to 2020. The empirical results show that digitalization level has positive effect on both exploratory innovation and exploitative innovation, and the effect on exploitative innovation is stronger than on exploratory innovation. Moreover, the moderating effect of industrial knowledge base is significant on “digitalization–exploratory innovation” but not on “digitalization–exploitative innovation” relationship. By doing so, this study refines the research on the relationship between digitalization and firm innovation, and confirms that the usage of digitalization may lead to achieve an ambidextrous situation. This study also provides a theoretical basis for industrial differences of the effectiveness of digitalization, suggesting firms considering industrial characteristics to implement digitalization-assisted innovation practices.

## Introduction

According to the Digital China Development Report in 2020, China’s total digital economy has jumped to the second place in the world. As an infrastructure of society, digital technology has a profound impact on people’s production and life. Firms operating in digital economy are faced with ever-changing and highly competitive environment, thus most firms are seeking digital transformation to largely introduce digital technologies into firm production practice (Mariani and Matarazzo, 2021; Dixit et al., 2022). Digitization emphasizes the revolution of business logic content, business model reconstruction and industrial transformation and upgrading through digital technology (Hanelt et al., 2021). Thus, digitalization is not only about technological change (Tabrizi et al., 2019), but more importantly, the reshaping of firms’ knowledge structure involving cognitive and thinking revolution, which affects industries and firms’ creation of knowledge through different cognition and reasoning patterns (Nonaka, 1994; Asheim and Coenen, 2005; Pocol et al., 2022). Firms’ development in the context of digital economy is more driven by big data and digital culture (Martínez-Caro et al., 2020). However, the coverage of digital technologies is uneven in different industries and regions. And the key to the role of digital technology lies in whether firms can integrate new digital technologies with the real economy, and ultimately achieve firm innovation.

The new technological revolution triggered by digital technologies (e.g., big data, cloud computing, artificial intelligence, blockchain, etc.) has not only changed the firms’ cognitive state toward knowledge, management paradigm, and production and operation process of firms, but also changed the innovation model of firms (McAfee and Brynjolfsson, 2012; Scuotto et al., 2017; Annarelli et al., 2021; Giudice et al., 2021; Chin et al., 2022). From the practice, digital technologies have fundamentally transformed traditional firms and industries, thus raising emerging research topic to question the underlying assumptions and themes in innovation management. Recent studies have paid attention to the critical role of digital technologies in the launching of firms’ innovation (Cenamor et al., 2019; Ferreira et al., 2019), and some related researches have begun to explore the nature of innovation and incorporate new theoretical perspectives and constructs that reflect the various contexts in which the digitalization changes the consequences of subsequent innovation activities (Autant-Bernard et al., 2006; Lyytinen et al., 2016). In nature, different types of innovations involve different methods to combine and integrate knowledge into different operational process (e.g., process optimization or R&D and product upgrading) (Castro et al., 2011; Zahra et al., 2020; Hanelt et al., 2021; Wade and Vochozka, 2021). These lead to two types of innovation: exploratory innovation and exploitative innovation (March, 1991). In traditional business environment, many firms are unable to develop ambidextrous innovations due to limited resources, thus they must choose between an exploitative or exploratory innovation (Zhou et al., 2021). However, firms operating in today’s digital economy may face more dynamic and competitive environment, which push firms to introduce new technology (e.g., digital technology) to realize operational efficiency and mass production, and discovering new business opportunities at the same time, and gradually achieve an ambidextrous situation (Cenamor et al., 2019; Ferreira et al., 2019; Duan et al., 2021; Dixit et al., 2022; Huang et al., 2022; Xie et al., 2022). Researches note that topic on the relationship between digitalization and firm ambidextrous innovation is promising but remains largely unexplored, and call for more attention about this issue (Nambisan et al., 2017; Kuester et al., 2018). This study bases on the knowledge-based view and seeks to address such research topic and bridge the research gap in existing literatures by exploring the effect of digitization on firm ambidextrous innovation.

Some studies state that whether firms could benefit from digitalization varies across industries (e.g., Zhou et al., 2021). Some find that digitalization has mixed consequences under different business conditions, therefore researchers need to better understand contingence factors to further explain why digitalization makes different contributions to firm across industries. Since innovation bases on the integration of knowledge (Vǎtǎmǎnescu et al., 2022), from the industrial knowledge base theory, firms from different industries have different knowledge bases, thus have certain differences in the way of thinking, knowledge demands, and knowledge integration approach to carry out and trigger innovation (Asheim and Coenen, 2005; Zahra et al., 2020). Knowledge base which indicates rationale cognition of knowledge determines the nature, representation and acquisition of firm-related knowledge and then affect the application method and application degree of firm digital technology, and the nature of a firm’s knowledge base fundamentally depends on the nature of knowledge base of the industry in which the firm is located. According to Asheim and Coenen (2005), industrial knowledge bases can be divided into two types: synthetic knowledge bases and analytical knowledge bases. However, existing research mostly suggests that of digitization’s improvement and optimization on firm processes and operations (Zhou et al., 2021; Dixit et al., 2022), and rarely explores how firms’ knowledge integration methods affect the effectiveness of digitization. Therefore, it is necessary to consider the moderating role of industrial knowledge base to explain the industrial differences.

To sum up, existing studies have largely demonstrated that digitalization may enable firms to take ambidextrous innovations, and pointed out that such effect varies across industries, since innovation in different industries relies on various level of cognitive and reasoning of knowledge. However, little research has been done to explore the industrial difference in such context. This study focuses on two research questions: (1) could digitization enable firms’ ambidextrous innovation? And (2) how such relationships vary across industries? To address these two research questions, this study incorporates knowledge-based view to explore the effect of digitization level on firm ambidextrous innovation, and examine the contingency effect of a crucial industrial level factor—industrial knowledge base. By doing so, this study makes significant contributions to the existing literatures. First, this study deepens our understanding of the effect of digitalization on different innovation types from the knowledge-based view. The empirical results show that digitization level has a positive effect on both exploratory and exploitative innovation, and the impact on exploitative innovation is stronger than that on exploratory innovation. The results indicate that digitization may enable firms to integrate knowledge throughout different operational process to realize exploratory innovation and exploitative innovation at the same time. Second, this study extends the industry level boundary conditions on the effectiveness of digitalization. The findings show the moderating effect of industrial knowledge base on the relationship between digitalization level and firm exploratory innovation but not on the relationship between digitalization level and exploitative innovation, which partly suggesting that the effect of digitization varies in synthetic knowledge-based industries and analytical knowledge-based industries.

This paper is organized as follows. The next section presents the relevant literature review and then develop the conceptual model with testable hypotheses. Then the methodology including the data and sampling are described. In the next section, this paper summarizes the results of empirical results, and then concludes the research findings in the following section. The discussion part concludes the theoretical and managerial implications, limitations and future research directions.

## Literature review

### Exploratory/exploitative innovation and ambidexterity

Faced with high uncertain environment and fierce market competition, innovation is becoming increasingly important for firms’ survival and growth in today’s digital economy (Dixit et al., 2022). Innovations may take different forms such as upgrades, modifications, and extensions of the existing IT product, products new to the firm or to the market, the region and even new to the society (Li and Atuahene-Gima, 2001). March (1991) differentiates two types of basic innovation due to the degree of innovation: exploration and exploitation. A firm’s innovation can emphasize exploration or exploitation innovation strategy, one, or both (March, 1991; Gedajlovic et al., 2012).

Firms demonstrating exploratory innovation usually actively seek for radical changes, collect fresh resources and expand aggressively to generate innovations that significantly transfer existing products and services. Such firms are commonly engaged in seeking for new opportunities, including search, discovery, experimentation, risk-taking decisions, and proactive activities (March, 1991; Cheng and Van de Ven, 1996). Correspondingly, exploratory innovations require not only new resources and knowledge or departure from existing resources and knowledge, but also new methods to integrate and utilize these knowledge to offer new designs, develop new distribution channels, and introduce differentiated product or service (Abernathy and Clark, 1985; Vǎtǎmǎnescu et al., 2022), and are aimed at satisfying the emerging and dynamic needs of customers or markets, or anticipating new potential market needs. Naturally, returns associated with exploratory innovation are more variable and distant in time (He and Wong, 2004). These exploratory-oriented firms are often specialized in the creation of new capabilities and are adaptive to respond to the turbulent environmental changes (Gupta et al., 2006), but a firm’s overemphasizing of exploration increases firms’ risk to failing to appropriate returns because of its costly search and experimentation activities (Cao et al., 2009).

Firms preferring to exploitative orientation often possess highly refined routines that leverage clearly identified core competitive advantages (March, 1991; Mikalef et al., 2020) and launch innovations that refine and reinforce existing products and services to meet the needs of existing customers or markets, through combing and recombining of existing resources at hand to broaden existing knowledge and skills, improve established designs, expand existing products and services, and increase the efficiency of existing distribution channels (Abernathy and Clark, 1985). Thus, returns related to exploitative innovation strategy are certain and closer in time (He and Wong, 2004; Cao et al., 2009), but in the highly changing and competitive environment, exploitative innovation may be unsustainable to some degree.

To sum up, regarding scarce resources, capabilities and limited attention (He and Wong, 2004), firms usually need to get a balance between the exportation of new opportunities and the exploitation of existing capabilities (March, 1991; Levinthal and March, 1993; Cao et al., 2009; Chin et al., 2020; Mikalef et al., 2020) because the two innovation strategies often compete for same scarce resources and place somewhat conflicting demands on organizational processes, structures, and cultures (March, 1991). As concluded above, both exploratory and exploitative innovation have assets and liabilities for firms. Thus, some innovation scholars emphasize the overwhelming importance of simultaneously pursuing exploratory and exploitative innovation, and see a balance of the two as central to the notion of organizational ambidexterity (Tushman and O’Reilly, 1996; O’Reilly and Tushman, 2008; Cao et al., 2009; Chin et al., 2020). Researches demonstrate that firms must simultaneously strive for a dual innovation strategy termed as “ambidextrous innovation” in order to pursue sustainable development, as such dual strategies facilitate the balance between short- and long- term performance (March, 1991; He and Wong, 2004).

A lot of prior literatures on ambidexterity defends the complementarities that exist between the exploratory and exploitative innovation orientations (Tushman and O’Reilly, 1996; O’Reilly and Tushman, 2008; Cao et al., 2009). Specifically, ambidexterity can positively affect firm performance by allowing firms to achieve both efficiency and effectiveness through new opportunity discoveries in the dynamic environment. However, these orientations require different organizational structures and related resources, so that a lot of firms pursuing ambidexterity fail (O’Reilly and Tushman, 2008), especially for those firms which are faced with severe resource constrains. For this matter, some related researches suggest that ambidexterity may be an impossible or ineffective goal, suggesting that firms would benefit more from focusing on either exploitation or exploration. However, recent studies suggest that the introduction of new technology (e.g., digital technology) may help firms realize such ambidextrous situation (Cenamor et al., 2019; Ferreira et al., 2019; Dixit et al., 2022; Kraus et al., 2022). Next, we will explore how digitalization affects firm ambidextrous innovation.

### Digitalization level and firm ambidextrous innovation

Digitalization involves the usage of digital technology to create value in new ways to boost fundamental firm change (Hanelt et al., 2021; Zhou et al., 2021; Dixit et al., 2022; Kraus et al., 2022). In the digital economy, digital technologies are a key enabler of firm innovation (Scuotto et al., 2017; Urbinati et al., 2020; Giudice et al., 2021; Suler et al., 2021; Bin, 2022). More and more firms are trying to seek new opportunities for innovation and development in the ever-changing market environment through digital transformation, so as to achieve overtaking on corners and gain sustainable competitive advantages (Richard and Devinney, 2005; Dean and Kretschmer, 2007; Yoo et al., 2010; Lyytinen et al., 2016). For most firms, digitization is the use of digital technology to improve the infrastructure of the firm (Canhoto et al., 2016; Pagani and Pardo, 2017; Hopkins and Siekelova, 2021). Digital innovation can also be used to describe, whether totally or partially, the results of innovation (Iansiti and Lakhani, 2014; Porter and Heppelmann, 2014, 2015; Nambisan et al., 2017). The mature application of digital technology continuously empowers the transformation and upgrading of traditional industries, and fosters new industries, new formats, and new models (Cenamor et al., 2019; Ferreira et al., 2019; Annarelli et al., 2021; Xie et al., 2022). Expanding the level of digitalization into firms’ operation involves utilizing digital technologies (e.g., big data, cloud computing, artificial intelligence, blockchain, etc.) to represent, process, store, and communicate the broadest possible range of information, resources, knowledge, and valuable data (Tilson et al., 2010; Yoo et al., 2010, 2012; Ardito et al., 2021; Usai et al., 2021; Wade and Vochozka, 2021; Chin et al., 2022).

Digitalization has led researchers to question increasingly the explanatory powers and utility of existing conceptualizations of innovation (Yoo et al., 2012; Cenamor et al., 2019; Annarelli et al., 2021; Xie et al., 2022). Existing literatures hold different views on whether to be digital or not (Ferreira et al., 2019; Ardito et al., 2021; Usai et al., 2021). A large amount of studies have reached an agreement and recognized the important impact of digitalization and firm innovation. Related research combines the theory of dynamic capability, absorptive capacity theory, and organizational resilience theory to reveal the mechanism of digitalization on firm innovation (Yoo et al., 2012; Cenamor et al., 2019; Eller et al., 2020; Nasiri et al., 2020). Most of these studies found that digital market products and digital business process innovation require certain digital-related capabilities (Eller et al., 2020; Nasiri et al., 2020; Appio et al., 2021), and the combination of digital capabilities and digital orientation can help firms innovate, to ensure the mass production more efficiently and also contribute to the upgrade of products/service (Hopkins and Siekelova, 2021). As a result, firms with a broader or deeper level of digital technology implementation can introduce more radical innovations, thereby better leveraging the potential value of their existing technologies at hand. In general, digital technologies are catalysts for product innovation and become generative resources that expand the space for product or service offerings (Blichfeldt and Faullant, 2021).

Some other studies find that effectiveness of digitalization to the performance varies across firms and industries. Zhou et al. (2021) points out that current research on digitalization has produced mixed findings regarding its outcomes in different contexts, therefore researchers need to better understand the contingency factors to further uncover under what conditions digitalization can contribute to firm. Their study focuses on service industry and finds out that digitalization alone could not help improve firm service performance such as enhancing customer communication, maintaining long-term relationships with customers. Specially, digitalization could only contribute to the performance for those with high level of entrepreneurial orientation and relatively small firm assets. This line of research leads the researches to think more about industrial differences when considering the effectiveness of digitization.

### Industrial differences in firm innovation: Knowledge-based view

A number of researches suggest that digital technologies have fundamentally transformed traditional firms and industries (Cenamor et al., 2019; Ferreira et al., 2019; Dixit et al., 2022). But most of these researches focus on exploring the firm-level differences (e.g., Zhou et al., 2021). For example, some scholars have begun to explore the boundaries of digitalization affecting firm innovation, mainly focusing on some firm level characteristic such as environment orientation, entrepreneurial orientation and firm assets size (Ardito et al., 2021; Usai et al., 2021; Zhou et al., 2021), and leave the industry-level difference related research underdeveloped (Appio et al., 2021).

Recently, some studies have begun to pay attention to industry differences and believe that there are significant differences in the impact of digitalization levels on firm innovation across different industries (e.g., Zhou et al., 2021; Dixit et al., 2022; Kraus et al., 2022). The main reason is that the penetration rate of digitalization across industries is different. There are industry differences in both the need for digital technology and the ability to utilize digital technology (Ferreira et al., 2019). On the other hand, the knowledge bases and innovation models required for firm innovation in different industries are quite different. Existing research believes that industry differences affect the effect of digitalization on firm innovation. At the same time, because the knowledge base affects the way and approach of firm innovation, it also affects the effect of digitalization. Therefore, it is necessary to deeply explore the relationship between digitalization and firm innovation from the perspective of industrial knowledge base.

Industrial knowledge base indicates that the basic professional information or knowledge that can be shared and shared among the same type of knowledge creation organizations in the industry, mainly including synthetic knowledge base and analytical knowledge base (Asheim and Coenen, 2005). Synthetic knowledge-based industries are industries where innovation occurs primarily through existing knowledge or new combinations of knowledge, that is, primarily the application or recombination of existing knowledge, through designing or creating something for a functional purpose (Moodysson et al., 2008). Synthetic knowledge base mainly exists in industries that re-research and combine to a certain extent with existing knowledge, such as machinery industry, food manufacturing, equipment engineering installation. In those industries, knowledge is mainly tacit and needs to communicate face-to-face due to the characteristics of difficulty in paper recording and codification. Through this way, knowledge utilization and application usually lead to incremental innovation. An analytical knowledge base refers to industrial settings, where scientific knowledge is of great importance, and where knowledge creation is based on the cognitive and reasoning industries of scientific knowledge. In other words, analytical industrial knowledge bases mostly exist in industries that produce new knowledge based on advanced scientific principles (e.g., IT and bio-tech) (Hanelt et al., 2021; Wade and Vochozka, 2021; Pocol et al., 2022). Most of the knowledge in such industry exists in research institutes, laboratories, universities and other related R&D departments in a dominant state that is easy to spread. This type of knowledge base requires a large investment of time, energy and intelligence, and may lead to radical innovation through intense knowledge creation (Consoli and Elche-Hortelano, 2010).

In sum, industrial knowledge base indicates an industry’s rationale cognition of knowledge. A synthetic knowledge base is primarily about innovation through the generation of new knowledge through scientific research, and analytical knowledge base refers to the understanding and interpretation of characteristics of the (natural) world using natural systems and following scientific laws. The typical differences between two types of industrial knowledge base are presented in the following table (see Table 1).

**TABLE 1:** Typical differences between two types of industrial knowledge base.

	Synthetic knowledge base	Analytical knowledge base
Knowledge content	Large-scale, tacit knowledge	Professional, explicit knowledge
Knowledge types	Technical knowledge	Scientific knowledge
How knowledge is created	Induction	Deduction
Forms of knowledge creation	Know-how, practical skills	Patents or publications
Knowledge rationale cognition	Application/combination of existing knowledge	Following scientific laws to develop new knowledge
Example of industry	Machinery	Biomedicine

Existing researches on industrial knowledge base mainly focus on the influence of industrial knowledge base on the construction of regional innovation system and innovation network (Asheim and Coenen, 2005, 2006; Gertler and Wolfe, 2005). In the firm-level related area, existing studies mostly utilize industrial knowledge theories to explain why and how companies innovate, but few related studies combine industrial knowledge theories with knowledge-based views to explore the important role of different industrial knowledge bases to explore the industrial differences in the effectiveness of firm digitalization.

## Theoretical framework and hypothesis

Innovation is essentially the creation of knowledge, and innovation activities must follow the unique nature and integration of knowledge (Castro et al., 2011). Considering the nature of innovation, some scholars propose the knowledge-based view (KBV) (Grant, 1996), and suggest that the knowledge possessed by firms is the source of innovation and the key to obtain sustainable competitive advantages (Miller and Shamsie, 1996; Johnson et al., 2002; Dean and Kretschmer, 2007). As Nonaka (1994) stated, knowledge is a cognitive state or cognitive fact, and cognition is derived from a state of understanding gained through experience and learning, and thus knowledge can also be described as the extent and sum of knowing, discovering, and learning. Usually, firms leverage differentiated knowledge from their internal employees, or through integrating knowledge that may be ignored or which exists outside the organization to generate new knowledge (Barley et al., 2018; Zahra et al., 2020). Thus, firms can expand their knowledge base internally or externally (Laursen and Salter, 2006; Escribano et al., 2009). Notwithstanding, a large piece of research is devoted to explore how external factors (e.g., development of new technology) may affect the innovation processes [see Galende (2006)], leaving aside the internal heterogeneity (e.g., firms’ ability to use new technology) that shapes the innovation dynamic. For this matter, even though the basic link between firms’ digitalization and innovation is on the whole persuasive, more remains to be explored and unfolded about its detailed and complicated nature from the knowledge-based view.

A number of researches base on knowledge management and organizational learning related literatures and offer an integrative framework to explain how and why firms launch innovation (Grant, 1996; Barley et al., 2018). In digital economy, firms are operating in ever-changing and competitive environment, and the technology continues to evolve and accelerate the need of fresh knowledge and new approach to integrate and rationale of such knowledge, which demonstrates the progressive primacy of knowledge-intensive industries (Dixit et al., 2022). Hence, in such new competitive landscape, firms should give increasingly attention and energy to knowledge and intellectual assets, recognizing that new knowledge and learning, and its effective implementation are key antecedents to achieve and maintain a competitive position (Galende, 2006), and one of the most effective ways comes directly from continuous innovations through the employment of new technology. Furthermore, firms’ innovation process in the digital economy is more than a knowledge job, and thus the innovation is the most knowledge-intensive business activities, then firms should depend very closely on the specific knowledge the firms possess, as well as on firms’ ability to integrate and deploy such knowledge (Nonaka and Takeuchi, 1995; Consoli and Elche-Hortelano, 2010; Huang et al., 2022).

Digitization greatly improves firms’ efficiency and effectiveness through collecting and processing a huge amount of knowledge and data. It can link product design, production, marketing and feedback, make quick responses to market demands, and speed up the pace of innovation of new products (Wade and Vochozka, 2021); also, digitization could enable firms to realize mass production (Hopkins and Siekelova, 2021; Suler et al., 2021), optimize business processes, improve operational efficiency, reduce costs and increase customer value (Gavrila and Ancillo, 2020). As such, existing studies agree that digitization helps firms innovate through effectively integrating internal and external resources and managing the innovation process more efficiently and effectively.

There are usually two different types of firm innovation activities: exploratory innovation and exploitative innovation. Exploratory innovation is a large-scale, radical innovation with the intention of seeking new possibilities; exploitative innovation is a small-scale, incremental innovation with the intention of improving the *status quo* (March, 1991). Existing studies have shown that digitization has a significant impact on firm innovation, but few have differentiated its effects on exploratory and exploitative innovation. Considering that different innovation activities have different demands on the firms’ knowledge integration, this study will discuss the impact of firm digitization on exploratory innovation and exploitative innovation, respectively.

### Digitalization level and firm ambidextrous innovation

Innovation initiatives in the digital economy have attracted interest from researchers and practitioners primarily because of the corresponding economic and social influence (Heirman and Clarysse, 2007; Kuester et al., 2018). March’s (1991) notions of exploration and exploitation are used as the basis for their conceptualization of a firm’s innovation strategy (Gedajlovic et al., 2012), and a firm’s innovation can emphasize exploratory or exploitative innovation, one, or both (March, 1991; Gedajlovic et al., 2012). However, exploratory innovation and exploitative innovation somehow competes for limited resources and requires different structures, thus some research shows that ambidexterity may be an impossible or ineffective goal, suggesting that firms would benefit more from focusing on either exploitative innovation or exploratory innovation (Zhou et al., 2021). However, recent studies suggest that the introduction of digital technology may accelerate firms’ realization of ambidextrous innovation (Cenamor et al., 2019; Ferreira et al., 2019). Thus, this study holds the view that digitalization level would help firms launch both exploratory innovation and exploitative innovation.

For exploratory innovation, firms would utilize digital technologies to collect big data and develop big data analysis capability to process the huge amount of data and transform these into valuable “small” data (e.g., knowledge and resources) (Cenamor et al., 2019). In this way, firms can broaden the channels to acquire new and fresh knowledge and also could excavate more specialized and specific knowledge into technological innovation (Mariani and Matarazzo, 2021; Huang et al., 2022). For example, firms can analysis and anticipate customers through contentiously dealing with dynamic sales data or could even understand consumer sentiment through text analysis on the social media. Following this way, firms can require new resources and knowledge to generate innovations that significantly reform current products and services, and it is highly possible for firms to meet the changing needs of emerging customers (Levinthal and March, 1993). In sum, exploratory innovation requires higher professional knowledge support and more significant innovation effects, and digital technology may help initiative exploratory innovation through acquisition of big data and new knowledge (Cenamor et al., 2019; Duan et al., 2021; Huang et al., 2022).

For exploitative innovation, firms may introduce digital technologies to leverage existing strengths, and build on existing business processes to optimize the process, realize mass production to promote delivery capability, and finally improve efficiency (Carugati et al., 2020; Mikalef et al., 2020; Hopkins and Siekelova, 2021). Specially, usage of digital technologies into the operational processes could help firms refine and expand the firms’ the existing capabilities, technologies, and paradigms (March, 1991; Ardito et al., 2021; Usai et al., 2021). Digital technologies may also empower the firms’ physical products or production systems, thus helps improve product functions and upgrade products to smart products, thereby improving the quality of products or services. Therefore, digitization could improve firms’ efficiency (such as shortening the production and delivery cycle) and the effectiveness (such as optimizing products) (Wade and Vochozka, 2021). In sum, digitalization and digital transformation may help improve the process innovation and improve the efficiency, and exploitative innovation can be achieved through infrastructure improvement, content reorganization and arrangement (Yang et al., 2021). Therefore, this study proposes that:

H1a: Digitization level facilitates exploratory innovation.

H1b: Digitization level facilitates exploitative innovation.

### Exploring the industrial differences

Innovation is the process of knowledge generation, diffusion and utilization, in which knowledge base plays a key role. Accordingly, the nature of industrial knowledge base affects the development of innovation activities. As mentioned above, industrial knowledge bases are divided into synthetic knowledge bases and analytical knowledge bases, and these two knowledge bases are defined according to the attributes of knowledge creation activities. Synthetic knowledge bases emphasize the application or recombination of existing knowledge. Industries with synthetic knowledge bases are mostly machinery, shipbuilding, computer development, and analytical knowledge bases are new knowledge generated through scientific research, including industries such as biomedicine, life sciences (Moodysson et al., 2008).

Industrial knowledge base refers that the firms located in those industries use different methods to integrate and create knowledge (Asheim and Coenen, 2005). Tacit knowledge is more important for synthetic knowledge-based firms because it is generated from relevant experience, and empirical knowledge is acquired in learning and communication and has a high popularity and is relatively simple and easy to circulate and spread. Analytical knowledge bases are more focused on explicit knowledge, have high scientific standards, and require long-term research and accumulation to obtain.

Specifically, firms operating in synthetic knowledge-based industries need to broad their knowledge sources and integrate a large amount of knowledge in the process of innovation activities (Tilson et al., 2010; Yoo et al., 2012), and use of digital technology could largely enable firms to gain the broadest possible range of information, resources, knowledge (Yoo et al., 2010). For firms operating in analytic knowledge-based industries, the innovation activities need the interpretation of knowledge, thus the promotion effect of digitalization in such scenario is relatively weak. Therefore, this study proposes that the industrial knowledge base moderates the relationship between digitization and firm innovation.

### The moderating role of industrial knowledge base on “digitalization level—exploratory innovation” relationship

In synthetic knowledge-based industries, such as aviation, automotive design, or semiconductor industries, a large amount of knowledge is accumulated and recombined, thus the application of digital technology may improve firms’ big data processing capabilities (Asheim and Coenen, 2005; Yoo et al., 2010). That is to say, the synthetic industrial knowledge base can be better combined with digital technology due to the stability, breadth and universality of its knowledge, so as to realize complex innovation and promote the exploratory innovation of firms (Dougherty and Dunne, 2012).

In comparation, most of the analytical knowledge-based industries face extremely complex and difficult-to-control technologies (Asheim and Coenen, 2005). Firms located in such industries usually follows scientific laws and invest large amount of effort into R&D, such complexity of knowledge-based innovation in analytical industries far exceeds the complexity of digital computing or big data analysis (Annarelli et al., 2021; Dixit et al., 2022). Therefore, this study proposes that:

H2a: Industrial knowledge base moderates the “digitalization level-exploratory innovation” relationship. Compared with the analytical industrial knowledge base, the synthetic industrial knowledge base has a stronger moderating effect on the relationship between digitalization level and firm exploratory innovation.

### The moderating role of industrial knowledge base on “digitalization level—exploitative innovation” relationship

Based on the knowledge-based view, the value of knowledge can be fully utilized when the knowledge base of a firm matches the corresponding technology. Innovations in synthetic knowledge-based industries often involve the use of new methods to improve production efficiency, product quality, or reliability (Asheim and Coenen, 2005; Castro et al., 2011). These innovations are mostly presented as improvements to existing products or processes. Therefore, in a synthetic knowledge-based industries, firms are more likely to take full advantage of digital technologies to improve their operational efficiency (Cao et al., 2009; Cenamor et al., 2019; Ferreira et al., 2019). However, in the analytical knowledge-based industries, firms usually launch new products or new production processes through intense R&D and knowledge creation (Asheim and Coenen, 2005). Therefore, in such industries, firms’ existing technologies and corresponding information systems could support firms to operate efficiently, thus the positive effect of digitization on exploitative innovation may be weakened. As suggested above, we propose that digitalization could enable firms to initiate exploitative innovation, and the synthetic knowledge base may be more beneficial for firms to utilize digital technology to improve the operation efficiency, thereby promoting firm exploitative innovation. Therefore, this study proposes that:

H2b: Industrial knowledge base moderates the “digitalization level-exploitative innovation” relationship. Compared with the analytical industrial knowledge base, the synthetic industrial knowledge base has a stronger moderating effect on the relationship between digitalization level and firm exploitative innovation.

**FIGURE 1:**
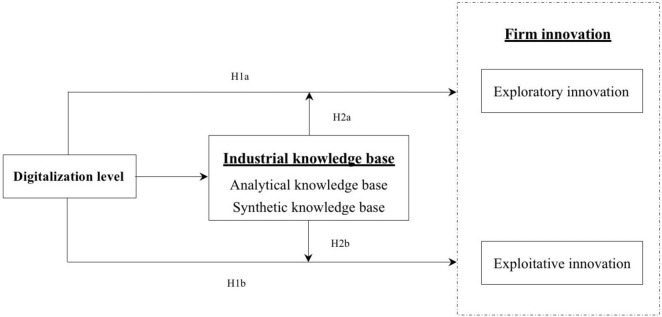
Conceptual model.

Figure 1 shows the conceptual model.

## Methodology

### Sampling and data

This study explores the effects of digitalization and firm ambidextrous innovation, and mainly focuses on the moderating effect of industrial knowledge base. This study uses secondary data to classify the industries to indicate the industrial knowledge bases to test the proposed hypotheses. According to the High-tech Industry Statistical Classification Catalogue issued by the National Bureau of Statistics, the IT industry and communication equipment manufacturing industry are selected as samples with a synthetic knowledge base, and the pharmaceutical manufacturing industry and chemical manufacturing industry are selected as research samples with an analytical knowledge base. This paper selects these four types of industries mainly for the following reasons: firstly, these types of industries started early, with strong technological foundation and innovation capability, and have good stability; It is very different from the core products. In the research, it can highlight the differences between industries and ensure the objectivity and operability of the research. Initially this study obtained a sample data of more than 400 listed companies in the above four industries from 2014 to 2020, and leave 394 sample companies after preliminary screening.

### Measurements

#### Independent variable: Digitalization level

According to previous research, this paper uses the method of keyword “search-matching-summation” to describe the level of firms’ digitalization using python crawler text recognition function. This article refers to the relevant reports of the G20, the Organization for Economic Cooperation and Development, and the United Nations, and summarizes the related literature on digital technology, and concludes the keywords involved in the application of digital technology including: Digitalization, Digital Transformation, Digital Technology, Digital Platform, Big Data, Data Analysis, Data Mining, Cloud Computing, Cloud Platform, Cloud Service, Artificial Intelligence, Blockchain, Internet of Things, 5G, Machine Deep Learning, Virtual Reality, Internet Plus, Industrial Internet (UNCTAD, 2017, 2019). This study uses Python to conduct text mining on the annual reports of listed companies, and forms variables of digitalization level of each company according to the frequency of digital-related words.

#### Dependent variable: Firm innovation

Following previous research, this study uses the effectiveness of patent to measure firm innovation. Patents are divided into three categories: invention patents, utility model patents and design patents. Prior researchers suggest that compared with utility model and design patents, invention patents propose brand-new technical solutions for products, methods with higher technical content and higher innovation value. Therefore, based on previous researches, this study measures firm exploratory innovation (Explore) using the total number of firm’s invention patents, and measures firm exploitative innovation (Exploit) using the total number of firm’s utility model and appearance patents.

#### Moderating variables: Industrial knowledge base

Industrial knowledge base could be divided into two types: synthetic knowledge base and analytical knowledge base. This study analyzes the knowledge base of various industries based on the existing literature, and focuses on the IT industry and the communication equipment manufacturing industry as the industry representatives of the synthetic knowledge base, and select the pharmaceutical manufacturing industry and the chemical manufacturing industry as the industry representatives of the analytical knowledge base. Industrial knowledge base was operated using a dummy variable: synthetic knowledge base was coded with a value of 0, and analytical knowledge base was coded with a value of 1.

#### Control variables

Referring to previous researches on digitalization and firm innovation, this study also controls some firm-level variables: firm age (Age), firm total assets (Asset), firm operating income (Income), and firm size (Size).

Table 2 presents the description of all the key variables.

**TABLE 2:** Description of key variables.

Name of variables	Symbol	Operation of variables
Digitalization level	DIG	The logarithm of the digital word frequency + 1
Exploratory innovation	Explore	The logarithm of the total number of firm’s invention patents + 1
Exploitative innovation	Exploit	The logarithm of total number of the firm’s utility model and appearance patents + 1
Industrial knowledge base	IKB	Dummy (0 = synthetic knowledge base, 1 = analytical knowledge base)
Founding year	Age	The logarithm of the firm’s founding year
Total asset	Asset	The logarithm of the firm’s total assets in 2020
Operating income	Income	The logarithm of the firm’s operating income in 2020
Firm size	Size	The logarithm of the firm’s employee number in 2020

## Results

This section presents the results for the hierarchical linear regression analysis. Table 3 presents the means, standard deviations, and bivariate correlations for the variables.

**TABLE 3:** Descriptive statistics and correlations matrix (*N* = 394).

Variables	Mean	SD	1	2	3	4	5	6	7	8
1 Dig	3.4032	1.74157	–							
2 Explore	2.5507	1.86116	0.455**	–						
3 Exploit	2.7979	2.17072	0.495**	0.712**	–					
4 IKB	0.505	0.5006	–0.580*	–0.221**	–0.431**	–				
5 Age	3.1613	0.28889	0.048	–0.005	–0.010	0.097	–			
6 Asset	4.2192	1.32597	0.296**	0.325**	0.289**	–0.009	0.249**	–		
7 Income	3.6123	1.50681	0.262**	0.322**	0.283**	0.036	0.157**	0.881**	–	
8 Size	7.9921	1.26791	0.296**	0.375**	0.338**	0.007	0.278*	0.847**	0.839**	–

SD, standard deviation. **Correlation is significant at the 0.01 level (2-tailed). *Correlation is significant at the 0.05 level (2-tailed).

We establish the following four equation models testing the relationship between digitalization level and firm innovation. Equations 1, 2, respectively, builds the relationship between digitalization level and firm exploratory/exploitative innovation, and Equations 3, 4 are to test the moderating effect of industrial knowledge base on digitization and firm exploratory/exploitative innovation.


(1) $$E\,x\,p\,l\,o\,r\,e=\alpha_{0}+\alpha_{1}\,D\,i\,g+\alpha_{2}\,A\,g\,e+\alpha_{3}\,A\,s\,s\,e\,t+\alpha_{4}\,I\,n\,c\,o\,m\,e+\alpha_{5}\,S\,i\,z\,e+\varepsilon$$

(2) $$E\,x\,p\,l\,o\,i\,t=\alpha_{0}+\alpha_{1}\,D\,i\,g+\alpha_{2}\,A\,g\,e+\alpha_{3}\,A\,s\,s\,e\,t+\alpha_{4}\,I\,n\,c\,o\,m\,e+\alpha_{5}\,S\,i\,z\,e+\varepsilon$$

(3) $$E\,x\,p\,l\,o\,r\,e=\gamma_{0}+\gamma_{1}\,D\,i\,g+\gamma_{2}\,A\,g\,e+\gamma_{3}\,A\,s\,s\,e\,t+\gamma_{4}\,I\,n\,c\,o\,m\,e+\gamma_{5}\,S\,i\,z\,e+\gamma_{6}\,I\,K\,B+\gamma_{7}\,D\,i\,g^{*}\,I\,K\,B+\varepsilon$$

(4) $$E\,x\,p\,l\,o\,i\,t=\beta_{0}+\beta_{1}\,D\,i\,g+\beta_{2}\,A\,g\,e+\beta_{3}\,A\,s\,s\,e\,t+\beta_{4}\,I\,n\,c\,o\,m\,e+\beta_{5}\,S\,i\,z\,e+\beta_{6}\,I\,K\,B+\beta_{7}\,D\,i\,g^{*}\,I\,K\,B+\varepsilon$$

To test the hypotheses, this study employs hierarchical linear regression and uses the entry approach and centralization variables to avoid multicollinearity. Table 4 shows the regression results.

**TABLE 4:** Results of hierarchical regression analysis^a^.

	Exploratory innovation			Exploitative innovation		
Variables	M1	M2	M3	M6	M7	M8
**Controls**						
Age	–0.115* (–2.387)	–0.090^†^ (–1.995)	–0.087* (–1.975)	–0.111** (–2.271)	–0.081^†^ (–1.836)	–0.061 (–1.424)
Asset	0.046 (0.420)	–0.032 (–0.315)	–0.017 (–0.173)	0.044 (0.393)	–0.047 (–0.464)	–0.069 (–0.709)
Income	–0.067 (–0.617)	–0.029 (–0.292)	–0.024 (–0.243)	–0.093 (–0.848)	–0.050 (–0.502)	–0.003 (–0.031)
Size	0.391*** (4.015)	0.323*** (3.568)	0.306** (3.427)	0.379*** (3.835)	0.301** (3.354)	0.328*** (3.773)
**Independent variable**						
Dig		0.365*** (7.988)	0.545*** (7.600)		0.423*** (9.347)	0.246*** (3.531)
**Moderator**						
IKB			0.189** (2.625)			–0.285*** (–4.061)
**Interaction**						
Dig*IKB			–0.246** (–4.082)			–0.002 (–0.030)
R^2^	0.142	0.265	0.295	0.118	0.281	0.332
AdjustedR^2^	0.134	0.255	0.283	0.109	0.272	0.320
R^2^ change	0.142	0.122	0.031	0.118	0.164	0.051
F change	15.993***	63.809***	8.333***	12.849***	87.360***	14.640***

^a^Reports standardized regression; t-values are given in parentheses. ^†^p < 0.1; *P < 0.05; **P < 0.01; ***P < 0.001.

In Table 4, M1, M2, and M3 test the exploratory innovation related hypothesis. M1 includes only control variables. Regarding control variables, the results show the non-significant effects of firm asset (β = 0.046, n.s.) and firm operational income (β = −0.067, n.s.). The results show that firm age has a significant negative effect on exploratory innovation (β = −0.111; *p* < 0.05), which suggest that mature firms are less motivated to innovate than new start-ups. The result also shows that firm size has a significant positive effect on exploratory innovation (β = 0.379, *p* = 0.000).

Among the four control variables, only firm size has a significant relationship with firm exploratory innovation (β = 0.391, *p* = 0.000). M2 tests the main effect of digitalization level on exploratory innovation. The addition of the main effect accounts for 12.2% of the variance in exploratory innovation over and above M1 (R^2^ change = 0.122, *p* = 0.000). And the effect of digitalization level on exploratory innovation is significant and positive (β = 0.545, *p* = 0.000). This result supports H1a. M3 contains the moderator and the interactive effect to test the moderating effect of industrial knowledge base between digitalization level and exploratory innovation. As Table 4 shows, the interaction term has a significant negative effect on exploratory innovation (β = −0.246, *p* = 0.000). The results indicate that compared with the analytical industrial knowledge base, the synthetic industrial knowledge base has a stronger moderating effect on the relationship between digitalization level and firm exploratory innovation. This result supports H2a.

The same steps are taken to verify the effects on firm exploitative innovation. M4 includes only control variables. Regarding control variables, the results show the non-significant effects of firm asset (β = 0.044, n.s.) and firm operational income (β = −0.093, n.s.). The results show that firm age has a significant negative effect on exploratory innovation (β = −0.115; *p* < 0.05). The result also shows that firm size has a significant positive effect on exploratory innovation (β = 0.391, *p* = 0.000).

**FIGURE 2:**
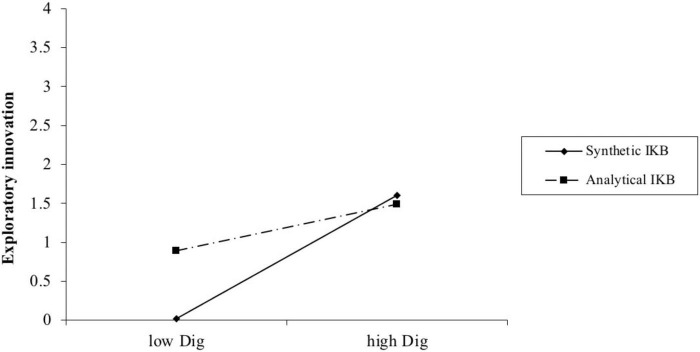
Industrial difference in the effect of digitalization on exploratory innovation.

Among the four control variables, only firm size has a significant relationship with firm exploitative innovation (β = 0.379, *p* = 0.000). M5 tests the main effect of digitalization level on exploitative innovation. The addition of the main effect accounts for 16.4% of the variance in exploratory innovation over and above M4 (R^2^ change = 0.164, *p* = 0.000). And the effect of digitalization level on exploitative innovation is significant and positive (β = 0.423, *p* = 0.000). This result supports H1b. To add interpretation, we plot the interaction effect in Figure 2 and find that it is in the expected direction. M6 contains the moderator and the interactive effect to test the moderating effect of industrial knowledge base between digitalization level and digitalization level. As Table 4 shows, the interaction term has no significant effect on digitalization level. Thus, H2b is not supported.

## Conclusion

This study focuses on two research questions: (1) could digitization enable a firm’s ambidextrous innovation? and (2) how such relationships vary according to different industries? This study introduces the knowledge-based view to try to explain the industrial differences and focuses on the moderating role industrial knowledge base between “digitalization—ambidextrous innovation” relationship. Based on data of listed companies from China’s four industries from 2014 to 2020, this study draws the following conclusions: first, aligning with most of prior related studies, this study suggests that digitalization has a positive impact on firm ambidextrous innovation. Specifically, the effect of digitization level on exploitative innovation is stronger than that on exploratory innovation (β = 0.423, *p* = 0.000; β = 0.365, *p* = 0.000). Compared with exploratory innovation, the barrier of exploitative innovation is lower, so firm could easily break through such barriers and use digital technology to enable exploitative innovation. Due to the difference in the degree and direction of innovation between exploratory innovation and exploitative innovation, firms will have certain differences in the resources input and integration of knowledge of such two types of firm innovations.

The result also shows that industrial knowledge base has a moderating effect on the relationship between digitization and firm exploratory innovation, but not on the relationship between digitization and firm exploitative innovation. Aligning with prior research, we suggest that innovation is the creation of knowledge, and firms in synthetic knowledge-based industries are mostly manifested as knowledge induction processes such as inspection, experimentation, computer simulation or time work, and less involved in knowledge deduction processes, so such firms can use digital technology in acquiring broader access to the knowledge sources required for innovation, and quickly integrating and summarizing knowledge into firm innovation. For firms operating in analytical knowledge-based industries, the innovation process starts from general scientific knowledge and derives individual and special knowledge through logical reasoning or inductive deduction. These results confirm both the industry difference and innovation type difference when exploring the effects of firms’ digitalization.

## Discussion

### Theoretical contributions

This study makes two key contributions to the existing literatures and theories. First, this study contributes to the emerging digitalization research by demonstrating the positive relationship between firm digitalization and ambidextrous innovation. Although a lot of studies emphasize that ambidexterity could help firms make full use of existing advantages, and also grasp the new opportunities and possibilities for further development (March, 1991), many firms pursuing ambidexterity fail because different innovation orientations compete for firms limited resources and usually require different organizational structures (Tushman and O’Reilly, 1996; O’Reilly and Tushman, 2008). Ambidexterity literatures have largely focused on the traditional business environment and investigated the antecedents of ambidextrous innovation from both external and internal perspectives (Gibson and Birkinshaw, 2004; Gedajlovic et al., 2012). In today’s digital economy, firms are mostly faced with highly dynamic and competitive environment, and the use of new technology may help firms realize ambidextrous innovation to better coping with external environment changes (Dixit et al., 2022), however, little empirical research has paid attention to the unique context of digital economy. This study responds to the call of prior research and base on knowledge-based view to explore whether digitalization could enable firm to realize ambidexterity. The empirical results show that digitalization facilitate firm to achieve an ambidextrous situation, and specifically, digitalization has differentiated effect on exploratory innovation and exploitative innovation. These findings deepen our understanding of the “digitalization—ambidextrous innovation” relationship (Ardito et al., 2021; Usai et al., 2021).

Second, this study extends the boundary research on the effectiveness of digitization level through examining the contingency effect of a crucial industrial level factor—industrial knowledge base. Some existing related studies have paid attention to the role of digitalization in assisting firm innovation, and moved a step further to exploring some firm-level factors, such as firm size and entrepreneurial orientation (Ardito et al., 2021; Usai et al., 2021; Zhou et al., 2021), but few studies have distinguished the industry difference in the effect of digitalization on firm innovation (Yoo et al., 2012; Cenamor et al., 2019; Ferreira et al., 2019). This study agrees that innovation is the combination and integration of knowledge (Grant, 1996), and industrial knowledge base is an important factor in determining the way firms integrate knowledge and significantly affects the relationship between digitization and firm innovation. Thus, this study combines knowledge-based view to deeply examine the moderating role of industrial knowledge base on the relationship between digitization level and firm innovation. The results show that industrial knowledge base has a moderating effect on “digitization—exploratory innovation” relationship, but not on “digitization—exploitative innovation” relationship. By doing so, this study further enriches the boundary research on the effectiveness of digitization from an industry difference perspective (Moodysson et al., 2008; Zhou et al., 2021).

### Managerial implications

This study has practical implications for firms’ digital practice in different industries and also for different innovation orientations. First, this study shows that firm digitization has positive effect on firms’ ambidextrous innovation, and has different effects on different types of firm innovation. Therefore, for firms that focus on innovation, they should pay attention to the implementation of digital technology to integrate knowledge to both improve the operational efficiency and also research and development in introducing and upgrading new products. Specially, digital technology can be utilized to link product design, production, marketing and feedback, make quick responses to market demands, and speed up the pace of innovation of new products; meanwhile, digital technology could also be used to optimize business processes, improve delivery efficiency, reduce costs, and increase customer value (Gavrila and Ancillo, 2020).

Second, this study suggests that firms from different industries should introduce different levels of digitization according to their requirements of innovation activities. In the process of digitalization, firms need to consider their industrial characteristics such as industrial knowledge base. Specifically, for firms conducting in industries with synthetic industrial knowledge bases such as machinery, shipbuilding, and communications, it is necessary to actively develop digitalization, build a digital layout for the whole operational processes, and improve the application scope and degree of digitalization, so as to enhance firms’ exploratory innovation capability.

### Limitations and future research directions

Based on prior literatures, this study further explores the industrial differences in the effect of digitalization on firm innovation from the perspective of industrial knowledge base. This is an exploratory study, and has some limitations, which leave some directions for future research. First, this study aims to test the industry differences of the effectiveness of digitalization, since different industries relies on various level of cognitive and reasoning of knowledge. Thus, this study introduces the moderating effects of industrial knowledge base. However, firm characteristics could also affect firm’s ways to percept and integrate knowledge, whereas industrial knowledge base couldn’t indicate firm variance. Future research could base on this study and further conjointly test both the industry- and firm- level contingency factors to extend such line of research. Second, based on prior related research, this study uses secondary data to measure the digitalization level and firm innovation. However, there may be a certain deviation from firms’ real digitalization level, and the implementation of innovation and the generation of innovation effects need to go through a certain period of time, and the effect of digitization on innovation is a gradual process. Future research could collect data through conducting longitudinal survey to capture the actual digitization level and firm innovation activities to in-depth exploration of underlying mechanism how and under what conditions digitalization would enhance firm innovation.

## Data availability statement

The original contributions presented in this study are included in the article/supplementary material, further inquiries can be directed to the corresponding author.

## Author contributions

QX and HL contributed to the conceptualization and results interpretation. YC contributed to the methodology, results interpretation, and reviewing and editing the manuscript. HL and KT contributed to the data collection, methodology, statistical analysis, and results interpretation. All authors contributed to the manuscript and approved the submitted version.
